# Effect of α-Synuclein Overexpression on NAPP-129 and TLQP-62 in Rat Brain and Plasma

**DOI:** 10.3390/medsci14020195

**Published:** 2026-04-13

**Authors:** Antonio Luigi Manai, Barbara Noli, Aqsa Anjum, Elias Manca, Maria Antonietta Casu, Marie-Christine Pardon, Cristina Cocco

**Affiliations:** 1 Department of Biomedical Sciences, University of Cagliari, 09042 Monserrato, CA, Italy; antonioluigi.manai@unica.it (A.L.M.); barbara.noli@unica.it (B.N.); anjumaqsa97@gmail.com (A.A.); manca.elias1@gmail.com (E.M.); 2 National Research Council-Institute of Translational Pharmacology, 09050 Pula, CA, Italy; mariaantonietta.casu@cnr.it; 3 School of Life Sciences, University of Nottingham, Nottingham NG7 2UH, UK; marie.pardon@nottingham.ac.uk

**Keywords:** VGF, Parkinson’s disease, biomarkers, neurodegeneration, α-synuclein

## Abstract

**Background:** In Parkinson’s disease (PD), changes in the brain begin before clinical symptoms. We have previously shown that VGF precursor levels were reduced in a presymptomatic PD animal model. **Objectives:** In the present study, we investigated whether two VGF precursor-derived products, namely NAPP-129 protein and TLQP-62 peptide, also exhibit alterations using the same PD animal model. **Methods:** Specifically, rats were unilaterally injected in the substantia nigra with a viral vector overexpressing green fluorescent protein (N = 12) or α-synuclein (N = 13), the latter resulting in mild dopaminergic alterations without overt motor deficits. **Results:** NAPP-129 and TLQP-62 were investigated in the substantia nigra, striatum, and plasma by Western blotting or immunoassays using specific antibodies against NAPP and TLQP sequences, alongside other NERP-1- and AQEE-related products. Plasma samples of a Huntington’s disease mouse model were also analyzed. We found reductions in NAPP-129 and TLQP-62 levels in the substantia nigra along with a decrease in NAPP- and TLQP-like plasma immunoreactivity in α-synuclein-overexpressed rats, while the striatum was not affected. NERP-1- and AQEE-related products were not altered. No changes were found in the Huntington’s disease model. **Conclusions:** These findings indicate that NAPP-129 and TLQP-62 may enhance the sensitivity and specificity of biomarker-based strategies for PD.

## 1. Introduction

Parkinson’s disease (PD) is a progressive neurodegenerative disorder characterized by the selective degeneration of dopaminergic neurons in the substantia nigra (SN), leading to impaired function of the nigrostriatal pathway and the development of motor symptoms typical of the disease [1,2,3]. The neuropathology of PD is characterized by the presence of Lewy bodies, which are intracellular inclusions mainly composed of misfolded α-synuclein (α-syn) [4,5]. Although α-syn represents a central pathological hallmark, methodological limitations have hindered its reliable use as a peripheral biomarker [6,7], and robust biological markers for the early diagnosis of PD are still lacking [8,9,10]. Increasing evidence indicates that the neurodegenerative process underlying PD begins many years before the appearance of motor symptoms [11]. This prodromal phase is characterized by subtle molecular alterations [12] and non-motor manifestations [13], highlighting an important window for early diagnosis and potential neuroprotective interventions. In this context, the early identification of biomarkers detectable in biological fluids represents a major priority in PD research. Among potential candidates, neuropeptides derived from the VGF (no acronym) precursor protein have attracted increasing attention in the context of neurodegenerative disorders. VGF (or proVGF) is a neurosecretory precursor protein undergoing extensive proteolytic processing leading to the production of numerous bioactive peptides/proteins, some of which are implicated in neuronal survival [14] and synaptic plasticity [15]. Importantly, distinct VGF-derived products appear to be differentially altered across neurodegenerative diseases. For example, specific VGF peptides are altered in Alzheimer’s disease, whereas a different set of peptides is affected in amyotrophic lateral sclerosis [16]. In PD, alterations appear to preferentially involve products originating from the C-terminal region of the VGF precursor. Reduced VGF levels, measured by ELISA using an antibody directed against the C-terminal region of human proVGF, have been detected in the plasma of PD patients at the time of diagnosis. These reductions persisted following short-term levodopa treatment but returned to control levels after long-term therapy [17]. Consistent with these findings, alterations in the C-terminal region of proVGF have been confirmed using mass spectrometry in urine [18] and cerebrospinal fluid (CSF) samples from PD patients [19,20]. Although the investigation of candidate PD biomarkers in patients remains crucial, preclinical studies are essential to elucidate the role and involvement of these candidates within nigrostriatal circuits, as well as to confirm their decreased expression in peripheral tissues. Indeed, preclinical studies further support the involvement of VGF C-terminal alterations observed in PD patients. In toxin-induced models of advanced PD, such as 6-hydroxydopamine [17] or fipronil exposure associated with motor impairment [21], marked alterations of VGF expression (using an antibody directed against the C-terminal region of rat/mouse proVGF) have been reported in the SN in association with severe nigral dopaminergic system neurodegeneration. More recently, using a presymptomatic PD model based on α-syn overexpression in the SN, we have shown that a small reduction in proVGF levels in both SN and peripheral blood (plasma) parallels mild nigral dopaminergic system alterations in the absence of overt motor deficits [22]. These findings suggest that VGF changes, especially those affecting the C-terminal region, may represent early molecular events associated with dopaminergic system dysfunction in nigrostriatal circuits. Based on this background, the present study selectively investigated two C-terminal proVGF-derived products, the TLQP-62 peptide and the NAPP-129 protein, whose names, like other VGF-derived products, reflect the N-terminal amino acid sequence followed by the peptide length. TLQP-62 peptide is one of the few VGF peptides with characterized bioactivity and has been shown to modulate synaptic plasticity and memory through the brain-derived neurotrophic factor signaling pathway, promoting dendritic growth and spine remodeling [23,24]. In contrast, the NAPP-129 protein has no defined biological function, although fragments containing the NAPP sequence have been reported to be reduced in the CSF of PD patients [20]. Therefore, using the enzyme-linked immunosorbent assay (ELISA) and/or Western blotting (WB), we investigated whether TLQP-62 and NAPP-129 are altered in SN and peripheral bood (plasma) in the same animal model, which shows mild dopaminergic system alterations and in which a decrease in full-length proVGF levels has been reported [22].

## 2. Materials and Methods

### 2.1. Adeno-Associated-Virus-Mediated Treatment in Experimental Animals

**Figure 1:**
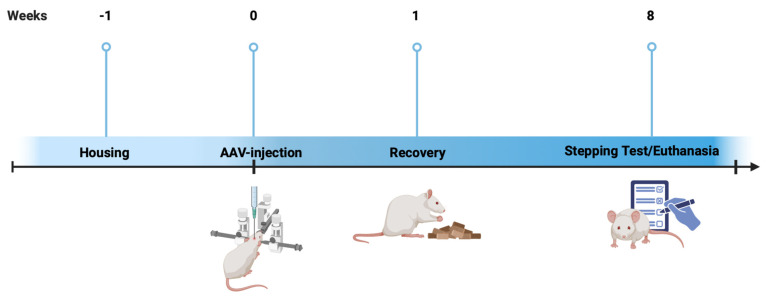
Timeline of the rat model. AAV (adeno-associated virus) was injected unilaterally into the SN (substantia nigra) to overexpress α-syn (alpha-synuclein) or GFP (green fluorescent protein).

Male Sprague–Dawley rats (275–300 g; Envigo, Italy) were used for adeno-associated virus 6 (AAV6) treatment. All animal procedures were conducted in accordance with the European Directive (EU 2010/63) and the Italian Legislative Decree (D.Lgs 26/2014), and they were approved by the local ethics committee and the Italian Ministry of Health (protocol no. 829/2019 PR). Rats were randomly allocated into two groups and received a unilateral injection into the SN of AAV-6 expressing either α-syn (AAV-α-syn; n = 13) or green fluorescent protein (AAV-GFP; n = 12). The AAV protocol was previously described [22]. Eight weeks after the transgene delivery, the animals were tested for their motor performance at the stepping test and for their general motor activity in an open field. After sacrifice, brain tissues were collected for ELISA and WB analyses, and blood samples were obtained for ELISA. Brain areas of interest were collected fresh and immediately snap-frozen on dry ice to preserve molecular integrity and stored at −80 °C until further processing. Blood samples were collected into ethylenediaminetetraacetic acid (EDTA)-coated tubes and centrifuged (4000× *g*, 10 min). The experimental timeline of the rat model is shown in Figure 1. We have previously demonstrated that this model closely replicates a presymptomatic stage of PD. Indeed, the locomotor activity test revealed no difference in the total distance traveled among the groups, and the same outcome was obtained when the rats were subjected to the stepping test, with no asymmetry detected in the forelimb use of AAV-α-syn animals compared to the GFP groups [22]. The absence of motor alterations was found to parallel the accumulation of pathological phosphorylated α-syn in the SN (but not in the striatum), with only modest thyrosine hydroxylase (TH) immunoreactivity reduction (~30%), associated with decreased proVGF levels in both brain (SN) and plasma [22]. The histochemical profile of the animal model is summarized in Table 1.

### 2.2. Huntington’s Disease Animals

Homozygous knock-in (Hdh^Q140/Q140^) Huntington’s disease (HD) mice expressing the yellow fluorescent protein (YFP; n = 3 males and n = 4 females) and their YFP littermates (Q140 n = 3 males and n = 5 females) were generated as previously described [25] in the University of Nottingham Bio Support Unit. Blood (approximately 200 μL) was drawn by cardiac puncture under terminal anesthesia and collected in a tube treated with EDTA (1.78 mg/mL). Plasma was then separated by centrifugation (3000× *g*, 10 min) at 4 °C, snap-frozen, and stored at −80 °C. All procedures were carried out in accordance with the UK Animals (Scientific Procedures) Act of 1986 under project license 40/3601 and approved by the University of Nottingham Ethical Review Committee.

### 2.3. VGF Antibodies

Through immunizations in rabbits, we produced the NAPP antibody against the NAPP-9 peptide (VGF_485–493_) (CPC Scientific, San Jose, CA, USA) conjugated at its N-terminus with keyhole limpet hemocyanin, the neuro-endocrine regulatory peptide-1 (NERP-1) antibody against the NERP-1 peptide (CPC Scientific, San Jose, CA, USA), and the AQEE antibody against the AQEE-10 peptide (VGF_586–595_) (CPC Scientific, San Jose, CA, USA). The TLQP-10 peptide was synthesized (CPC Scientific, San Jose, CA, USA) and conjugated at its C-terminus for immunizations in guinea pigs. All the antibodies were previously validated [26,27]. More specifically, validation of TLQP, AQEE, and NAPP antibodies [26] included: (i) affinity purification, performed by incubating each antibody with the corresponding immunogen antigen covalently immobilized on a sulfolink coupling resin; (ii) ELISA, in which each VGF antibody was tested against the appropriate antigenic peptide as well as against extended peptides differing in amino acid length, the latter showing low antibody reactivity; (iii) WB, using the pheochromocytoma-12 cell line (PC12) either expressing or lacking VGF, where a positive signal was detected only in cells expressing proVGF; and (iv) dot blotting, performed with synthetic NAPP-19, TLQP-62, and AQEE-30 peptides, which confirmed that each antibody specifically recognized its corresponding peptide without cross-reactivity with the other two. The immunoreactivity of the NERP-1 antibody for proteins in the 70–60 kDa range (corresponding to proVGF) as well as for peptides of approximately 1 kDa (corresponding to NERP-1) was validated in plasma and in neural stem cells (NSC-34) using WB and sephadex chromatography (coupled with ELISA), respectively [27]. In addition, WB analysis of the PC12 cell line confirmed the specificity of the NERP-1 antibody for VGF sequences, as a positive signal was observed only in cells expressing VGF [27].

### 2.4. Molecular Characterization

WB analyses were performed using SN samples from both the injected and contralateral sides of each AAV-α-syn-treated rat (6 samples per side, each obtained by pooling tissue from 2–3 animals) and AAV-GFP–treated rats (6 samples per side, each obtained by pooling tissue from 2 animals). Striatal samples from the same animals were analyzed individually (13 vs. 12 per side for AAV-α-syn vs. AAV-GFP–treated rats). Tissues were added to a 10 mL/g phosphate-buffered solution (PBS) plus protease inhibitor cocktail (Sigma, P8340, Darmstadt, Germany), 5 μL/mL (PBS: PO_4_ buffer, 0.01–0.05 M, pH 7.2–7.4 + sodium chloride (0.15 M)), and promptly homogenized with an ultraturrax for one minute. Tissues were kept on ice for 10 min. The samples were then boiled for another 10 min, cooled down, and centrifuged at 3000 rpm for 15 min at 4 °C. The supernatant was then collected and stored frozen until use. The bicinchoninic acid protein assay kit (Thermo Scientific, Waltham, MA, USA) was used to measure protein concentrations. Samples were diluted in sodium dodecyl sulfate-reducing loading buffer to ensure equal loading of 30 micrograms of protein, denatured by boiling for 5 min, and centrifuged for 5 min. Proteins were separated by gel electrophoresis using a precast polyacrylamide gradient gel (NuPAGE 4–12% Bis-Tris Mini protein gel, Thermo Fisher Scientific, Waltham, MA, USA) for 30 min at 200 volts, then transferred to a polyvinylidene difluoride membrane (Amersham Hybond-P, GE Healthcare, Buckinghamshire, UK) for 1 h at 20 volts. Membranes were then blocked with 5% bovine serum albumin (BSA) diluted in tris-buffered saline + 0.01% tween-20 (TBS-T) for one hour at room temperature and incubated overnight at 4 °C with either rabbit anti-VGF C-terminus [22] (1:2000), guinea pig anti-TLQP (1:1000), or rabbit anti-NAPP (1:1000) antibodies diluted in BSA 5% and TBS-T. The next day membranes were rinsed three times with TBS-T and incubated at room temperature for 1 h with the appropriate horseradish peroxidase-conjugated secondary antibody: either donkey anti-rabbit antibody (1:10,000; Jackson ImmunoResearch, West Grove, PA, USA) or donkey anti-sheep antibody (1:10,000; Jackson ImmunoResearch). Finally, the antigen–antibody reaction was revealed using the Thermo Scientific Pierce Enhanced Chemiluminescence WB substrate. The ImageQuant LAS 4000 was used to detect chemiluminescence. To perform accurate optical density (OD) analysis, the striatum and SN membranes were incubated with an anti-actin goat antibody (1:1000, Santa Cruz Biotechnology, Dallas, TX, USA) to normalize the signal of target proteins. When possible, band intensity was analyzed using Image Studio software 6.0 (LI-COR, Biosciences, Lincoln, NE, USA).

### 2.5. ELISA

Competitive ELISAs were performed with the plasma of AAV-α-syn-treated rats (N = 13) compared to AAV-GFP-treated rats (N = 12). The HD mouse plasma samples were also used (N = 7 vs. 8; Hdh^Q140/Q140^ and YFP, respectively). Striatal samples (from both injected and contralateral sides) were obtained from both AAV-α-syn rats (N = 13) or GFP rats (N = 12). ELISAs were carried out with the antibodies we have raised against the proVGF C-terminus and synthetic VGF peptides (see VGF antibodies). Multiwell plates (Nunc, Milan, Italy) were coated with synthetic peptides as C-terminus [22], TLQP-10 (15 pmol/mL), NAPP-9 (20 pmol/mL), NERP-1 (10 pmol/mL), and AQEE-10 (160 pmol/mL) overnight at 4 °C and treated with PBS (containing 9% normal serum from the secondary antibody donor species, 0.2 mg/mL sodium azide, and 1 mg/mL EDTA) for 2 h at room temperature. Primary incubations with rabbit anti-NAPP (1:60,000) and guinea pig anti-TLQP (1:5000), rabbit proVGF C-terminus (1:40,000), rabbit anti-NERP-1 (1:10,000), and rabbit anti-AQEE (1:8000) were carried out in duplicate, including serial standard dilutions in parallel with samples (1:10). Biotinylated anti-rabbit and anti-guinea-pig secondary antibodies (1:10,000; from Jackson Immunoresearch), streptavidin–peroxidase conjugate (1:10,000 Biospa, Milan, Italy), and tetramethylbenzidine (X-traKem-En-Tec, Taastrup, Denmark) as a substrate were used to reveal the positive labeling. The reaction was then stopped with hydrochloric acid (1 mol/L), and the optical density was measured at 450 nm using a multilabel plate reader (Chameleon: Hidex, Turku, Finland). The recovery of synthetic peptides added to plasma and striatum samples at extraction was >85%. Intra- and inter-assay coefficients of variation (CV1 and CV2) and detection limits (DLs) were obtained for each TLQP, NAPP, and AQEE assay [26], as well as for the NERP-1 assay [27].

### 2.6. Statistical Analyses

Statistical analyses were carried out using StatistiXL 1.10 and GraphPad v.9.5 software. For each experimental dataset, the normality of data distributions was assessed using the Shapiro–Wilk test (*p* < 0.05). Homogeneity of variances was evaluated using the F-test, and, accordingly, pooled or individual variances were applied to two-tailed Student’s *t*-tests. Based on the results of the Shapiro–Wilk test, parametric or non-parametric *t*-tests were conducted as appropriate.

## 3. Results

### 3.1. Reduced NAPP-129 and TLQP-62 in the SN of AAV-α-Syn Rats

**Figure 2:**
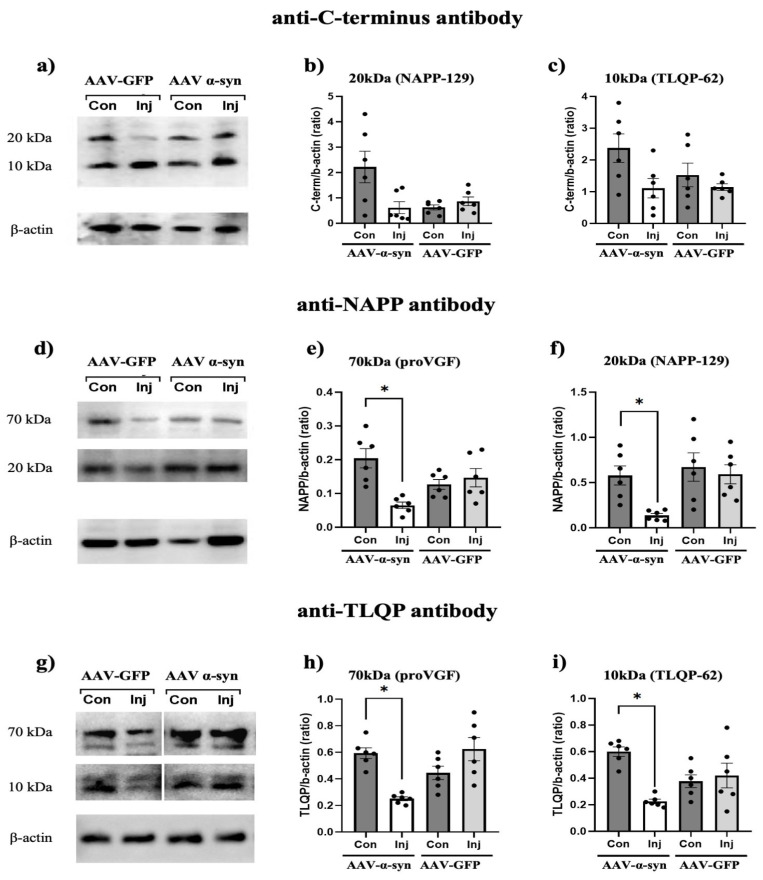
Nigral expression of NAPP-129 and TLQP-62 in AAV-rats. To assess NAPP-129 and TLQP-62 levels in the SN of AAV rats, WB analyses were performed using antibodies against the proVGF C-terminus, as well as the NAPP and TLQP sequences within proVGF (**a**–**i**). Using the antibody against the proVGF C-terminus, two immunoreactive bands were detected at approximately ~20 kDa (NAPP-129) and ~10 kDa (TLQP-62) in the four representative samples (**a**). Densitometric analysis showed no significant differences between rat groups for the ~20 kDa (**b**) nor the ~10 kDa (**c**) band, although a trend was observed. Using the NAPP antibody, two bands were labeled at approximately ~70 kDa and ~20 kDa, corresponding to proVGF and NAPP-129, in the four representative samples (**d**). Densitometric analysis showed a significant decrease in the ~70 kDa (**e**) and ~20 kDa (**f**) bands exclusively on the injected sides of the AAV-α-syn rats. Using the TLQP antibody, two bands were labeled at approximately ~70 kDa and ~10 kDa, corresponding to proVGF and TLQP-62, in the four representative samples (**g**). Densitometric analysis showed a significant decrease in the ~70 kDa (**h**) and ~10 kDa (**i**) bands exclusively on the injected sides of the AAV-α-syn rats. AAV: adeno-associated virus; α-syn: α-synuclein; GFP: green fluorescent protein; Con: contralateral; Inj: injected; (* *p* < 0.05). Densitometric analysis was done using pooled SN samples from both the injected and contralateral sides (6 samples per side, each obtained by pooling tissue from 2–3 animals) and AAV-GFP-treated rats (6 samples per side, each obtained by pooling tissue from 2 animals).

To reveal the NAPP-129 protein and the TLQP-62 peptide in SN samples using WB, we used antibodies directed against NAPP and TLQP sequences, as well as the antibody recognizing the proVGF C-terminus (Figure 2a–i). This latter antibody was selected because both NAPP-129 and TLQP-62 retain the C-terminal region of proVGF, as they are generated by upstream cleavage events. Using the antibody raised against the proVGF C-terminus sequence (Figure 2a), the following immunoreactive bands were consistently detected in SN samples from all AAV-treated rats: (i) a ~70 kDa band corresponding to proVGF [22], (ii) a ~20 kDa band corresponding to NAPP-129, and (iii) a ~10 kDa band corresponding to TLQP-62. Importantly, quantification using the VGF C-terminus antibody across AAV groups for the 20 and 10 kDa bands corresponding to NAPP-129 and TLQP-62 revealed that the signal intensities were not reduced, although a trend was observed (Figure 2b,c). When the anti-NAPP (Figure 2d) and anti-TLQP (Figure 2g) specific antibodies were used, the ~70 kDa band was detected by both antibodies, as expected, since NAPP and TLQP correspond to internal sequences of the proVGF precursor (see Supplementary Figure S1). Quantification of the ~70 kDa band signal using anti-NAPP (Figure 2e) and anti-TLQP (Figure 2h) antibodies revealed a significant comparable reduction (~30%; *p* < 0.05) on the injected sides of AAV-α-syn-treated rats compared with the contralateral sides, whereas no differences were observed in AAV-GFP-treated rats (comparing the two sides). These findings are consistent with our previous results obtained using the antibody directed against the proVGF C-terminus [22]. In addition to the ~70 kDa band, each antibody specifically recognized the band corresponding to its target, namely, NAPP-129 (~20 kDa, Figure 2d) or TLQP-62 (~10 kDa, Figure 2g). Quantification of both signal bands revealed a similar and significant comparable reduction (~30%; *p* < 0.05) in the AAV-α-syn-injected sides compared with the contralateral sides when labeled with the corresponding antibodies (Figure 2f,i). No such differences were observed between injected and control sides in AAV-GFP–treated rats. Hence, in response to PD-related pathology, not only proVGF but also two of its C-terminal-derived products are altered.

### 3.2. NAPP-129 and TLQP-62 Remained Unaltered in the Striatum of AAV Rats

**Figure 3:**
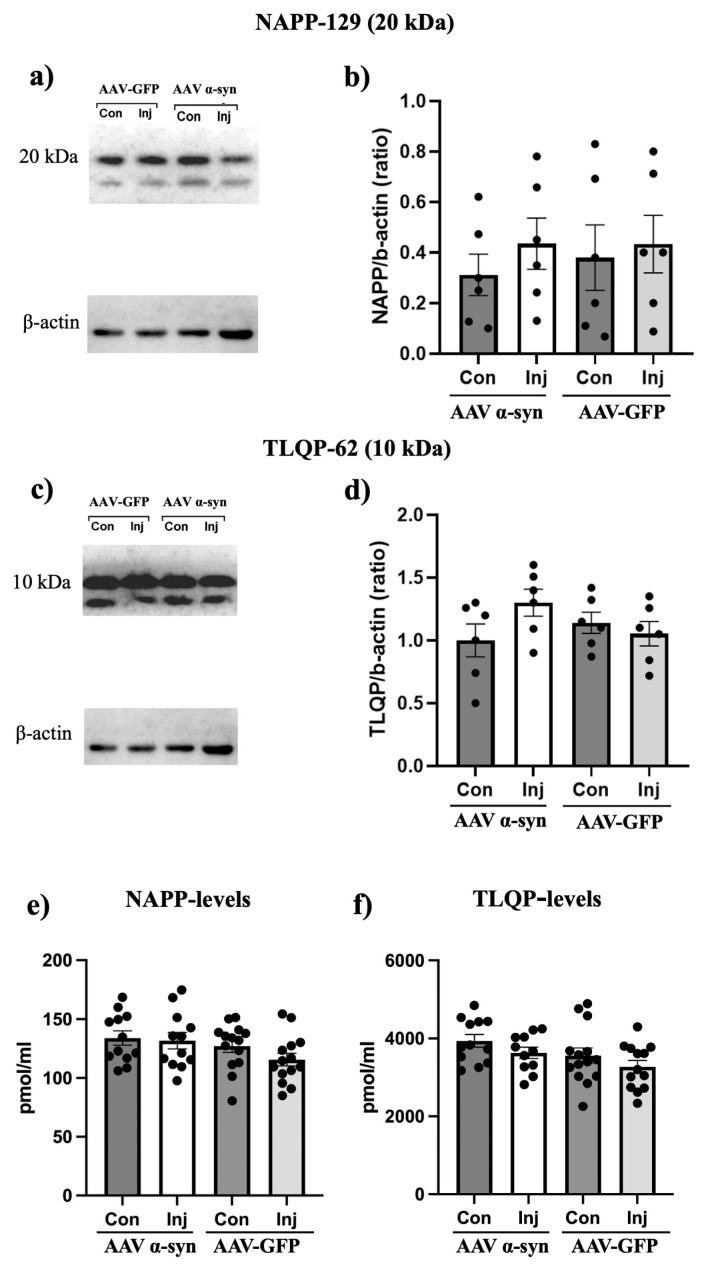
Striatal expression of NAPP-129 and TLQP-62 in AAV rats. To assess NAPP-129 and TLQP-62 in the striatum of the AAV rats, WB analyses were performed using antibodies against the NAPP and TLQP sequences within the proVGF (**a**–**d**). Using the NAPP antibody, one band was recognized at approximately ~20 kDa (corresponding to NAPP-129) in the four representative samples (**a**). Densitometric analysis of this band (**b**) did not reveal any changes between the different AAV rat groups (*p* > 0.05). Using the TLQP antibody, one band was recognized at approximately ~10 kDa, corresponding to TLQP-62, in the four representative samples (**c**). Densitometric analysis of this band (**d**) did not reveal any changes between the different AAV rat groups (*p* > 0.05). ELISAs using antibodies against NAPP (**e**) and TLQP (**f**) sequences revealed the absence of significant changes between the different AAV rat groups (*p* > 0.05). AAV: adeno-associated virus; α-syn: α-synuclein; GFP: green fluorescent protein; Con: contralateral; Inj.: injected; pmol/g tissue: picomoles/grams of tissue sample. Densitometric analysis was done using individual striatal samples (13 vs. 12 per side; for AAV-α-syn vs. AAV-GFP-treated rats).

Using striatal samples, we aimed to investigate differences in the NAPP-129 protein and TLQP-62 peptide by employing their respective antibodies and performing both WB and ELISAs (Figure 3a–f). The presence of NAPP-129 (Figure 3a) and TLQP-62 (Figure 3c) was confirmed by WB using the corresponding antibodies. No significant differences in NAPP-129 (Figure 3b) or TLQP-62 (Figure 3d) band intensity were detected between the AAV-α-syn rat groups or between the AAV-GFP groups (both *p* > 0.05). ELISA measurements with these antibodies further confirmed the lack of differences between the groups (Figure 3e,f; *p* > 0.05).

### 3.3. Reduced NAPP and TLQP Levels in the Plasma of AAV-α-Syn Rats

**Figure 4:**
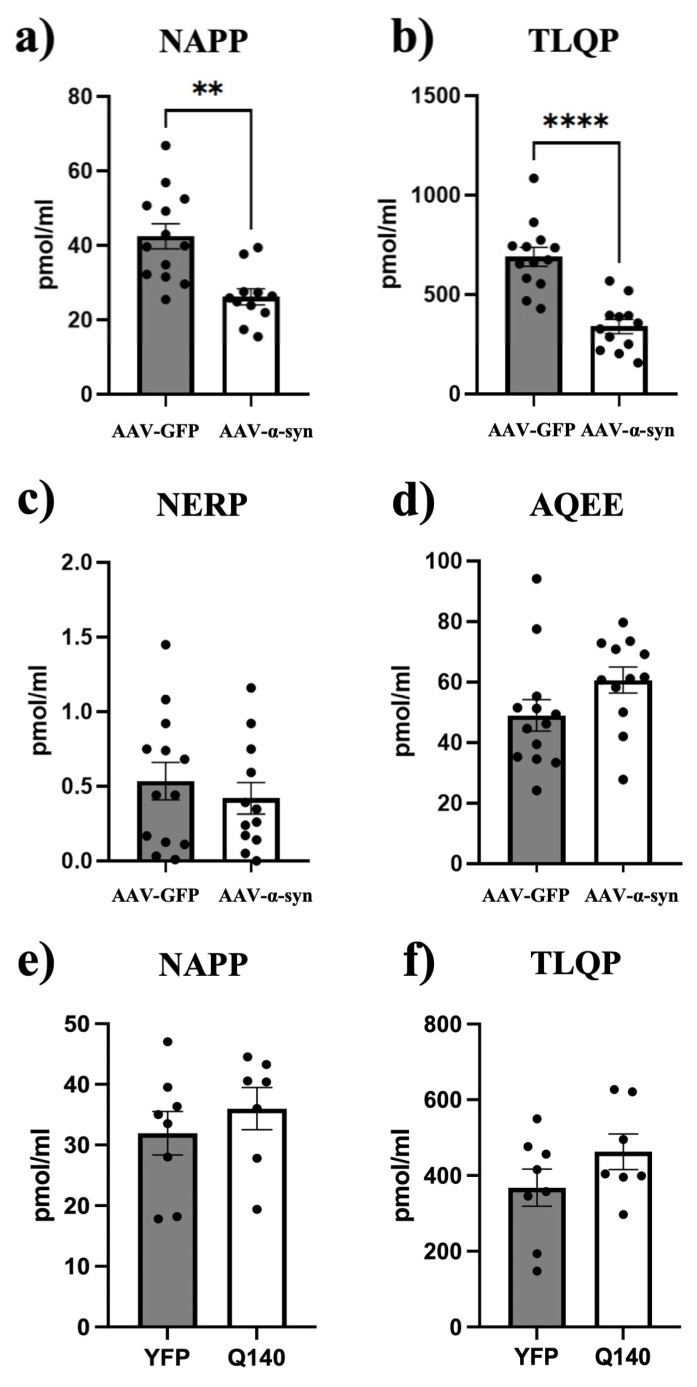
Plasma levels of the VGF-derived products. ELISAs were performed to quantify different VGF-derived products in plasma samples of AAV rats (13 vs. 12, AAV-α-syn vs. AAV-GFP: (**a**–**d**)). A decrease in NAPP (**a**) and TLQP (**b**) levels within AVV-α-syn rats compared to AAV-GFP rats was revealed (** *p* < 0.001 and **** *p* < 0.0001, respectively), associated with a lack of significant changes in the levels of NERP-1-related products (**c**) and AQEE levels (**d**) (both *p* > 0.05). AAV: adeno-associated virus; α-syn: α-synuclein; GFP: green fluorescent protein;. ELISA performed using NAPP (**e**) and TLQP (**f**) antibodies with samples from the Q140 Huntington mouse model and their YFP control littermates did not reveal any changes (both *p* > 0.05). Q140: homozygous knock-in (Hdh^Q140/Q140^); YFP: yellow fluorescent protein; pmol/mL: picomole/milliliter.

ELISAs using antibodies against VGF-derived products containing NAPP and TLQP sequences performed on plasma samples (Figure 4a–f) confirmed the results found in the SN by WB. Specifically, NAPP (Figure 4a) and TLQP (Figure 4b) levels showed a comparable 40–50% reduction (*p* < 0.001 and *p* < 0.0001, respectively) in response to AAV-α-syn. To determine whether the VGF reduction was also evident for other VGF-derived products, additional ELISAs targeting fragments containing the AQEE- and NERP-related sequences were performed; however, no significant differences were observed (*p* > 0.05: Figure 4c,d).

### 3.4. NAPP and TLQP Levels Were Unchanged in the Plasma of HD Mice

NAPP and TLQP assays performed on plasma samples from HD mice and their littermates revealed no significant differences between the groups for both markers (*p* > 0.05; Figure 4e,f).

## 4. Discussion

In the present study, we employed a preclinical model previously characterized as representing a presymptomatic stage of PD. In this model, the absence of motor deficits is accompanied by the accumulation of pathological phosphorylated α-syn aggregates in the SN, along with only modest dopaminergic alterations, as indicated by an approximately 30% reduction in TH-immunoreactivity [22]. We have previously shown that this model presented with a decrease in the full-length proVGF, detected by WB, both in the brain and in plasma. In the present study, we extend these findings by demonstrating that two specific C-terminal proVGF-derived products, the NAPP-129 protein and the TLQP-62 peptide, are also altered in the SN (but not in striatum) as well as possibly in the plasma (Supplementary Materials: Figure S2 and Table S1). The proVGF band in SN was consistently identified and reduced in AAV-treated rats when using antibodies recognizing the NAPP and TLQP sequences, in agreement with our previous observations obtained using a proVGF-directed antibody [22]. In contrast, reductions in the NAPP-129 and TLQP-62 bands were detected in SN only by their respective sequence-specific antibodies, demonstrating internal consistency, although this does not conclusively prove specificity. To our knowledge, our findings represent the first demonstration that specific VGF-derived products are altered in a presymptomatic PD model, highlighting the technical challenges involved in discriminating among closely related proVGF processing products. Nevertheless, several limitations should be acknowledged. First, overlaps in antibody recognition of different products with shared sequences cannot be excluded when using ELISA with plasma samples. Second, TLQP-62 and NAPP-129 were identified only by molecular weight, without sequence confirmation by complementary techniques, i.e., high-resolution spectrometry analysis. However, previous studies using spectrometry analysis have successfully identified multiple VGF products, including TLQP and NAPP sequences, in whole brains of control rats [21] and in plasma samples from healthy subjects [26]. Future studies will therefore aim to more precisely characterize the VGF-derived peptides/proteins altered in brain and peripheral biofluids by combining complementary analytical approaches. Interestingly, previous liquid chromatography–tandem mass spectrometry analyses have reported reduced levels of VGF C-terminal fragments, including peptides containing the NAPP sequence, in body fluids of patients with PD, such as CSF [19,20], which is in direct contact with the brain, suggesting that VGF decreases in body fluids reflect the biochemical and pathophysiological state of the brain. Accordingly, the hypothesis that peripheral VGF levels are closely associated with the brain’s dopaminergic status is strongly supported by evidence from both PD patients and animal models [17]. Specifically, VGF levels in PD patients were reduced at diagnosis and remained low after short-term levodopa treatment but increased to reach control levels following long-term therapy. A similar pattern was observed in advanced PD rat models, where VGF immunoreactivity (using a proVGF C-terminus antibody) in the SN disappeared after severe dopaminergic injury but was partially restored following levodopa treatment [17]. Consistent with this view, evidence from animal models further indicates that reductions in nigral TH immunoreactivity—whether severe (~60–90%) or moderate (~30%)—are consistently accompanied by proportional alterations in the VGF levels in both the SN and peripheral blood [17,22]. Notably, in our model, VGF-related alterations were region-specific and were not observed in the striatum, nor in HD animals. In our animal model, in contrast to the SN, the striatum displayed only moderate phosphorylated α-syn immunoreactivity, as previously reported [22], suggesting that pathological α-syn accumulation had not yet progressed sufficiently to induce detectable changes either in full-length proVGF or in its derived products, including NAPP-129 and TLQP-62. Moreover, not all proVGF-derived products were altered in this prodromal PD animal model. In particular, ELISA showed that the VGF-derived products containing NERP-1- and AQEE-related sequences were unchanged, indicating that proVGF processing is selectively affected rather than globally disrupted. These findings further support the concept that individual proVGF-derived products may reflect distinct neuropathological contexts and could respond differently, depending on disease stage or cellular stress conditions. Regarding the hypothesis explaining why these two VGF-derived products are reduced in PD, it can be proposed that NAPP-129 and TLQP-62 could be particularly sensitive to changes in the activity of proteases, including prohormone convertases, which are known to be involved in VGF processing [19,28]. Since α-syn aggregates are known to disrupt the secretory pathway [29], their reduction may reflect a broader impairment of peptide processing within the Golgi–endosomal network. Functionally, decreased peptide/protein availability could impair the vesicular sequestration of cytosolic dopamine, thereby increasing oxidative stress and promoting further α-syn misfolding. In conclusion, from a translational perspective, the combined assessment of total proVGF together with two C-terminal-derived products, such as the NAPP-129 protein and the TLQP-62 peptide, may enhance the sensitivity and specificity of biomarker-based strategies for PD. The use of multiple biologically related markers may be particularly advantageous for the early detection of PD, when pathological changes are subtle, and could also prove valuable for disease monitoring and therapeutic evaluation.

## Figures and Tables

**Table 1:** Immunohistochemical characteristics of the animal model.

		AAV-α-Syn			AAV-GFP			
	Staining	Contralateral	Injected	*p*-Value	Staining	Contralateral	Injected	*p*-Value
Stereology	TH	12,243 ± 464	8528 ± 1242	0.017	TH	11,345 ± 728	12,634 ± 865	0.658
	CH	52,099 ± 1950	47,642 ± 1425	0.095	CH	-	-	
OD	TH	22 ± 2	13 ± 1.8	0.0148	TH	25 ± 3.6	21 ± 2.6	0.7479
	VGF	96 ± 7.4	71 ± 16.2	0.0074	VGF	108 ± 6.4	101 ± 3.6	0.9086
	GAD65	144 ± 41	788 ± 39	<0.0001	GAD65	159 ± 19	144 ± 20	0.7640

AAV-α-syn: Adeno-associated virus expressing alpha-synuclein; AAV-GFP: Adeno-associated virus expressing green fluorescent protein, TH: Tyrosine hydroxylase, CH: Carazzi’s hematoxylin, OD: Optical density, VGF: No acronym, GAD65: Glutamic acid decarboxylase 65.

## Data Availability

The original contributions presented in this study are included in the article/Supplementary Materials. Further inquiries can be directed to the corresponding author.

## Supplementary Materials

The following supporting information can be downloaded at https://www.mdpi.com/article/10.3390/medsci14020195/s1. Figure S1: The figure illustrates the positions of NAPP-129 and TLQP-62 within proVGF protein. Figure S2: Original western blot membranes. Table S1: Statistical analysis.

## Abbreviations

AAV: Adeno-associated virus; AAV-GFP: Adeno-associated virus expressing green fluorescent protein; AAV-α-syn: Adeno-associated virus expressing alpha-synuclein; ANOVA: Analysis of variance; BSA: Bovine serum albumin; CSF: Cerebrospinal fluid; CV: Coefficient of variation; DL: Detection limit; EDTA: Ethylenediaminetetraacetic acid; ELISA: Enzyme-linked immunosorbent assay; EU: European Union; HD: Huntington’s disease; NERP-1: Neuroendocrine regulatory peptide-1; OD: Optical density; PBS: Phosphate-buffered saline; PC12: Pheochromocytoma-12 cell line; PD: Parkinson’s disease; SN: Substantia nigra; TBS-T: Tris-buffered saline with Tween-20; TH: Tyrosine hydroxylase; WB: Western blotting; YFP: Yellow fluorescent protein; α-syn: Alpha-synuclein.
