# Supplementary material for: Effect of α-Synuclein Overexpression on NAPP-129 and TLQP-62 in Rat Brain and Plasma
Source: Med Sci (Basel). 2026 Apr 13;14(2):195. doi: 10.3390/medsci14020195 (PMC13108048; doi:10.3390/medsci14020195)
Supplement: Supplementary file 1 [file medsci-14-00195-s001.zip › medsci-4137653-Supplementary.pdf]

Supplementary materials

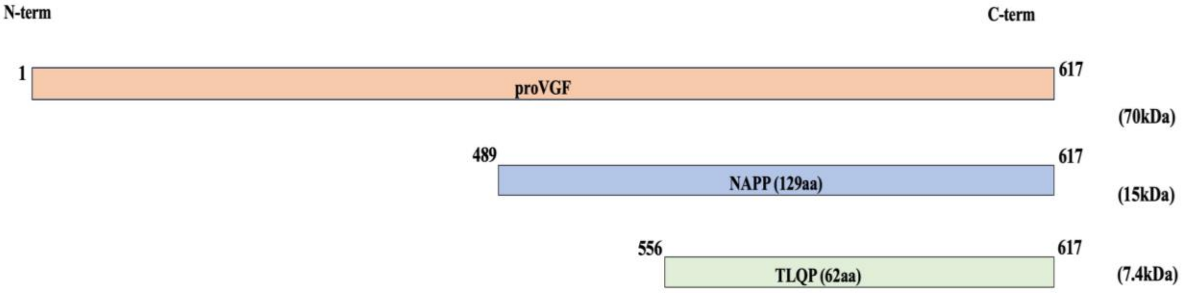

Figure S1. ProVGF and its products. ProVGF is a protein of 617 amino acids (approximately 70 kDa) that gives rise to several peptides, including the NAPP-129 protein ($VGF_{488–617}$; approximately 15 kDa) and the TLQP-62 peptide ($VGF_{556–617}$; approximately 7.4 kDa). NAPP-129 and TLQP-62 are named according to their N-terminal amino acid sequence, followed by their total length in amino acids.

Fig. 2, a anti-C-terminus antibody

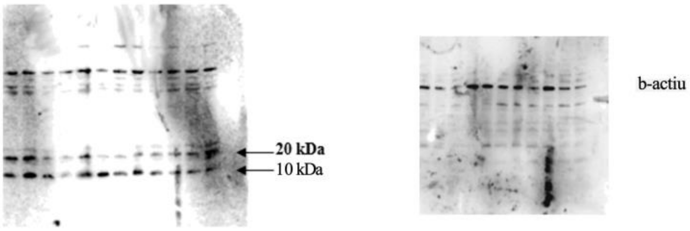

Fig. 2, d anti-NAPP antibody

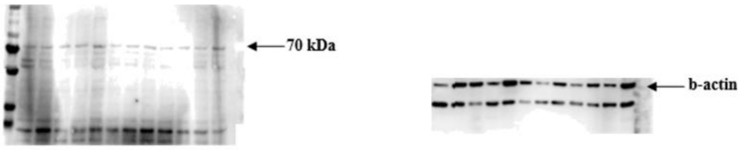

Fig. 2, g anti-TLQP antibody

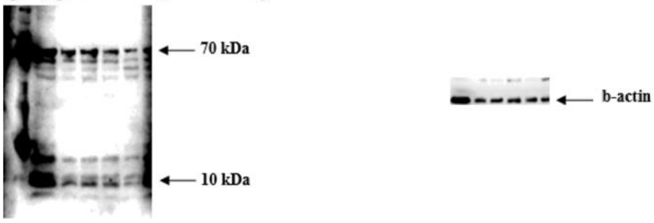

Fig. 3, b,d

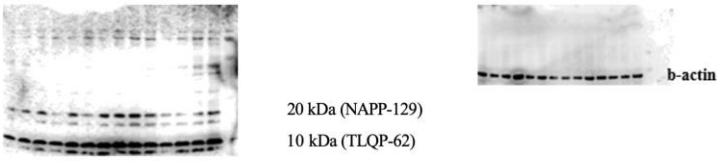

Figure S2. Original western blot membranes.

Table S1. Statistical analysis

Plasma – ELISA

	AAV GFP<br>Mean [SD], n.	AAV a-syn<br>Mean [SD], n.	Test type<br>[p value]	Confidence interval
**NAPP**	40.8 [13.3], 13	26.2 [7.3], 11	Welch's t [0.003]	-24.7 to -7.9
**TLQP**	670.6 [178.8], 13	336.3 [119.7], 12	Welch's t [0.0001]	-474.1 to -227.4
**AQEE**	48.9 [18.9], 13	60.6 [14.8], 12	Welch's t [0.1]	-2.3 to 25.7
**NERP**	0.53 [0.45], 13	0.42 [0.36], 12	Welch's t [0.48]	-0.45 to 0.22
	**YFP**<br>Mean [SD], n.	**Q140**<br>Mean [SD], n.		
**NAPP**	31.9 [10.2], 8	35.9 [9.2], 7	Welch's t [0.43]	-6.7 to 14.9
**TLQP**	367.9 [138.7], 8	462.7 [124.2], 7	Welch's t [0.19]	-51.8 to 241.4

Striatum – ELISA

	Control side<br>Mean [SD], n.	Injected side<br>Mean [SD], n.	Test type<br>[p value]	Confidence interval
**NAPP AAV GFP**	127.0 [19.6], 13	115.7 [20.5], 13	Student's t [0.15]	-26.9 to 4.2
**NAPP AAV a-syn**	134.1 [21.1], 12	131.7 [24.1], 12	Student's t [0.81]	-21.4 to 16.9
**TLQP AAV GFP**	3556 [745.4], 13	3274 [580.3], 12	Welch's t [0.37]	-820.8 to 321.4
**TLQP AAV a-syn**	3940 [561.7], 12	3634 [745.4], 11	Welch's t [0.17]	-762.5 to 150.3

Striatum – WB

	Control side<br>Mean [SD], n.	Injected side<br>Mean [SD], n.	Test type<br>[p value]	Confidence interval
**NAPP-129 AAV GFP**	0.37 [0.32], 13	0.40 [0.28], 13	Student's t [0.83]	-0.22 to 0.27
**NAPP-129 AAV a-syn**	0.32 [0.19], 12	0.45 [0.29], 12	Student's t [0.19]	-0.07 to 0.30
**TLQP-62 AAV GFP**	1.16 [0.43], 13	1.08 [0.43], 13	Student's t [0.65]	-0.42 to 0.27
**TLQP-62 AAV a-syn**	1.03 [0.35], 12	1.29 [0.31], 12	Student's t [0.08]	-0.01 to 0.54

Substantia nigra – WB

	Control side<br>Mean [SD], n.	Injected side<br>Mean [SD], n.	Test<br>[p value]	Confidence interval
**C-term (20 kDa) AAV GFP**	0.63 [0.24], 6	0.86 [0.42], 6	Student's t [0.25]	-0.19 to 0.68
**C-term (20 kDa) AAV a-syn**	2.22 [1.53], 6	0.62 [0.57], 6	Welch's t [0.06]	-3.2 to 0.003
**C-term (10 kDa) AAV GFP**	1.53 [0.91], 6	1.14 [0.26], 6	Welch's t [0.36]	-1.3 to 0.57
**C-term (10 kDa) AAV a-syn**	2.37 [1.1], 6	1.11 [0.75], 6	Welch's t [0.06]	-2.5 to -0.03
**NAPP (70 kDa) AAV GFP**	0.12 [0.04], 6	0.14 [0.06], 6	Student's t [0.01]	-0.04 to 0.09
**NAPP (70 kDa) AAV a-syn**	0.20 [0.07], 6	0.07 [0.02], 6	Welch's t [0.01]	-0.2 to -0.06
**NAPP (20 kDa) AAV GFP**	0.67 [0.38], 6	0.59 [0.26], 6	Student's t [0.68]	-0.5 to 0.34
**NAPP (20 kDa) AAV a-syn**	0.58 [0.26], 6	0.13 [0.05], 6	Welch's t [0.01]	-0.7 to -0.2
**TLQP (70 kDa) AAV GFP**	0.45 [0.12], 6	0.62 [0.21], 6	Student's t [0.11]	-0.04 to 0.4
**TLQP (70 kDa) AAV a-syn**	0.59 [0.10], 6	0.25 [0.03], 6	Welch's t [0.01]	-0.4 to -0.2
**TLQP (10 kDa) AAV GFP**	0.38 [0.12], 6	0.42 [0.23], 6	Student's t [0.68]	-0.18 to 0.28
**TLQP (10 kDa) AAV a-syn**	0.60 [0.08], 6	0.22 [0.04], 6	Student's t [0.01]	-0.46 to -0.28

Mean values are expressed in pmol/ml for ELISA; for densitometric analysis of the striatum and substantia nigra, the mean represents the ratio between VGF and β-actin.
